# A meta-analysis and trial sequential analysis comparing nonoperative versus operative management for uncomplicated appendicitis: a focus on randomized controlled trials

**DOI:** 10.1186/s13017-023-00531-6

**Published:** 2024-01-13

**Authors:** Francesco Brucchi, Greta Bracchetti, Paola Fugazzola, Jacopo Viganò, Claudia Filisetti, Luca Ansaloni, Francesca Dal Mas, Lorenzo Cobianchi, Piergiorgio Danelli

**Affiliations:** 1 University of Milan, Via Festa Del Perdono 7, 20122 Milan, Italy; 2 Unit of General Surgery I, Fondazione I.R.C.C.S. Policlinico San Matteo, Pavia, Italy; 3 University of Pavia, Corso Str. Nuova, 65, 27100 Pavia, Italy; 4 Division of General Surgery, Department of Biomedical and Clinical Sciences, University of Milan, L. Sacco University Hospital, 20157 Milan, Italy; 5 Department of Pediatric Surgery, Buzzi Children’s Hospital, 20154 Milan, Italy; 6 Department of Management, Università Ca’ Foscari, Dorsoduro 3246, 30123 Venezia, Italy; 7 Milan, Italy; 8 Collegium Medicum, University of Social Sciences, Łodz, Poland

**Keywords:** Acute appendicitis, Appendectomy, Antibiotic, Meta-analysis, Nonoperative treatment

## Abstract

**Background:**

The aim of this study is to provide a meta-analysis of randomized controlled trials (RCT) comparing conservative and surgical treatment in a population of adults with uncomplicated acute appendicitis.

**Methods:**

A systematic literature review was performed according to the Preferred Reporting Items for Systematic Reviews and Meta-Analyses (PRISMA) guidelines**.** A comprehensive search was conducted in MEDLINE, Embase, and CENTRAL. We have exclusively incorporated randomized controlled trials (RCTs). Studies involving participants with complicated appendicitis or children were excluded. The variables considered are as follows: treatment complications, complication-free treatment success at index admission and at 1 year follow-up, length of hospital stay (LOS), quality of life (QoL) and costs.

**Results:**

Eight RCTs involving 3213 participants (1615 antibiotics/1598 appendectomy) were included. There was no significant difference between the two treatments in terms of complication rates (RR = 0.66; 95% CI 0.61—1.04, *P* = 0.07, *I*^2^ = 69%). Antibiotics had a reduced treatment efficacy compared with appendectomy (RR = 0.80; 95% CI 0.71 to 0.90, *p* < 0.00001, *I*^2^ = 87%) and at 1 year was successful in 540 out of 837 (64.6%, RR = 0.69, 95% confidence interval 0.61 to 0.77, *p* < 0.00001, *I*^2^ = 81%) participants. There was no difference in LOS (mean difference − 0.58 days 95% confidence interval − 1.59 to 0.43, *p* = 0.26, *I*^2^ = 99%). The trial sequential analysis has revealed that, concerning the three primary outcomes, it is improbable that forthcoming RCTs will significantly alter the existing body of evidence.

**Conclusions:**

As further large-scale trials have been conducted, antibiotic therapy proved to be safe, less expensive, but also less effective than surgical treatment. In order to ensure well-informed decisions, further research is needed to explore patient preferences and quality of life outcomes.

**Supplementary Information:**

The online version contains supplementary material available at 10.1186/s13017-023-00531-6.

## Background

Acute appendicitis is a common abdominal emergency that requires prompt diagnosis and treatment. For over a century, open appendectomy was the only standard treatment for appendicitis. However, recent studies have challenged the necessity of surgery in uncomplicated cases of appendicitis, and nonoperative management (NOM) with antibiotics alone has emerged as a promising alternative [1–4]. Although appendectomy has long been considered the gold standard operative management (OM) for acute appendicitis, there is growing interest in NOM with antibiotics in both adults and children [5].

While nonoperative management may offer certain advantages over appendectomy, such as decreased morbidity and shorter recovery time, there are concerns regarding the efficacy and safety of this approach. For instance, nonoperative management may be associated with a higher rate of recurrent appendicitis and an increase in the duration of hospital stay [6]. Thus, it is important to evaluate the efficacy and safety of nonoperative management compared to appendectomy in uncomplicated cases of appendicitis.

Despite years of experience performing surgery to treat uncomplicated appendicitis, there is still a shortage of data that can be used to compare NOM and OM, making the choice between the two more challenging. This systematic review and meta-analysis of RCTs purpose was to compare NOM and OM in terms of efficacy, costs, length of hospital stay, quality of life and complications in a population of adults.

## Material and methods

A systematic literature review was performed according to the Preferred Reporting Items for Systematic Reviews and Meta-Analyses (PRISMA) guidelines and as outlined in a predefined protocol (PROSPERO 2023: CRD42023413780) [7].

### Literature search strategy

The PubMed, Scopus, and Cochrane Library databases and ClinicalTrials.gov, Google Scholar were screened without time restrictions up to November 23rd, 2023 using the Mesh major topic “appendicitis” and “surgery” and Mesh terms “appendectomy” and “conservative treatment”. The search query is available in the Additional file 1. Articles without free full text availability were searched through the University of Milan digital library in order to realize a complete research. The bibliographies of potentially relevant studies that were identified were manually searched for additional studies. Additionally, all studies that cited the primary studies were screened for inclusion on Google Scholar. We did not apply language or publication status restrictions.

### Eligibility criteria

The study selection criteria encompassed randomized controlled trials (RCTs) that investigated the comparison between antibiotic treatment and appendectomy in adult participants, presenting with uncomplicated acute appendicitis diagnosed either clinically or radiologically. Exclusion criteria consisted of non-randomized studies and studies that included patients with complicated appendicitis or children.

### Study selection

Two investigators (FB, GB) performed the literature search independently with the aid of Rayyan systematic review software [8]. Cases of disagreement were resolved by a third investigator (LC). In cases where multiple reports were found for the same study, data from all reports were utilized as necessary, while ensuring that there was no duplication of study participants.

### Data extraction

Data extraction was performed independently by two authors (F.B. and G.B.), with any discrepancies resolved through consultation with a third senior author (L.C.). Data were gathered and recorded in a digital database, including information on the baseline characteristics of the studies, including characteristics of patients as follows: exam blood test, Alvarado score [9], LOS, recurrence at 1 year, and efficacy of the treatment performed.

### Outcome measures

#### Primary outcome measures

1. Complication-free treatment success: the success of the initial treatment (nonoperative management or operative management) was evaluated based on an uncomplicated course, with no occurrence of postoperative complications (complications or recurrences for NOM; postoperative complications for surgical intervention)
2. Treatment efficacy based on 1-year follow-up: the efficacy of nonoperative management (NOM) was defined as achieving a definitive improvement without the need for surgery within a median follow-up of 1 year. Lack of efficacy in the NOM group included two scenarios: the persistence of acute appendicitis during hospitalization (referred to as index admission NOM failure, characterized by non-resolving appendicitis with persistent or worsening symptoms during the primary hospital stay) and recurrence of acute appendicitis. For OM, efficacy is defined as the resolution of symptoms following surgical treatment.
3. Postoperative complications: the analysis involved evaluating the number and rates of various postoperative complications.

#### Secondary outcome measures

1. The study analyzed the number and rates of patients treated with a laparoscopic approach in both groups.
2. Total costs: This encompassed the overall medical and surgical costs associated with the primary hospital stay.
3. Length of primary hospital stay: This refers to the number of days of inpatient admission during the initial hospitalization.
4. Quality of life following antibiotic therapy (AT) and surgical therapy (ST) was assessed.

### Assessment of risk of bias

To assess any potential bias in the studies included in the analysis, the researchers (F.B. and G.B.) utilized the risk of bias tool developed by the Cochrane Collaboration [10]. The studies were evaluated based on criteria such as selection bias, performance bias, detection bias, and attrition bias. A total risk of bias score was then determined based on these domains, with the levels categorized as low risk of bias, high risk of bias, or unclear risk of bias.

### Statistical analysis

Data from the individual eligible studies were entered into a spreadsheet for further analysis. Review Manager (RevMan) (Version 5.4.1. Copenhagen: The Nordic Cochrane Center, the Cochrane Collaboration, 2011). Risk Ratio (RR) was calculated for discrete variables with 95% confidence intervals (c.i.) calculated using a Mantel–Haenszel random-effects model. Mean Difference (MD) were calculated for continuous variables with 95% c.i. using an inverse-variance random-effects model. Statistical significance was taken at *P* < 0.05 using two-tailed testing. Heterogeneity among the trials was determined by means of the Cochrane Q value and quantified using the *I*^2^ inconsistency test [10].

### Trial sequential analysis

Cumulative meta-analyses of trials face a susceptibility to stochastic errors due to inadequate data and repetitive testing as the data accumulates [11, 12]. Trial sequential analysis (TSA) was employed, for primary outcome measures, to evaluate the statistical robustness of the data in a cumulative meta-analysis. TSA served as a means to gauge whether the existing evidence was sufficiently conclusive. The adjusted required information size (RIS) was computed using a significance level (alpha) of 0.05 (two sided) and a power (1—beta) of 0.20 (corresponding to 80% power). This calculation involved a control group proportion derived from the outcomes of our meta-analysis for binary outcomes. The decision to seek additional evidence from additional trials can be determined by assessing whether the cumulative Z-curve crosses trial sequential monitoring boundaries (TSMB) or the futility zone. Trial sequential analysis version 0.9 beta (http://www.ctu.dk/tsa) was used for all these analyses [13].

## Results

**Fig. 1:**
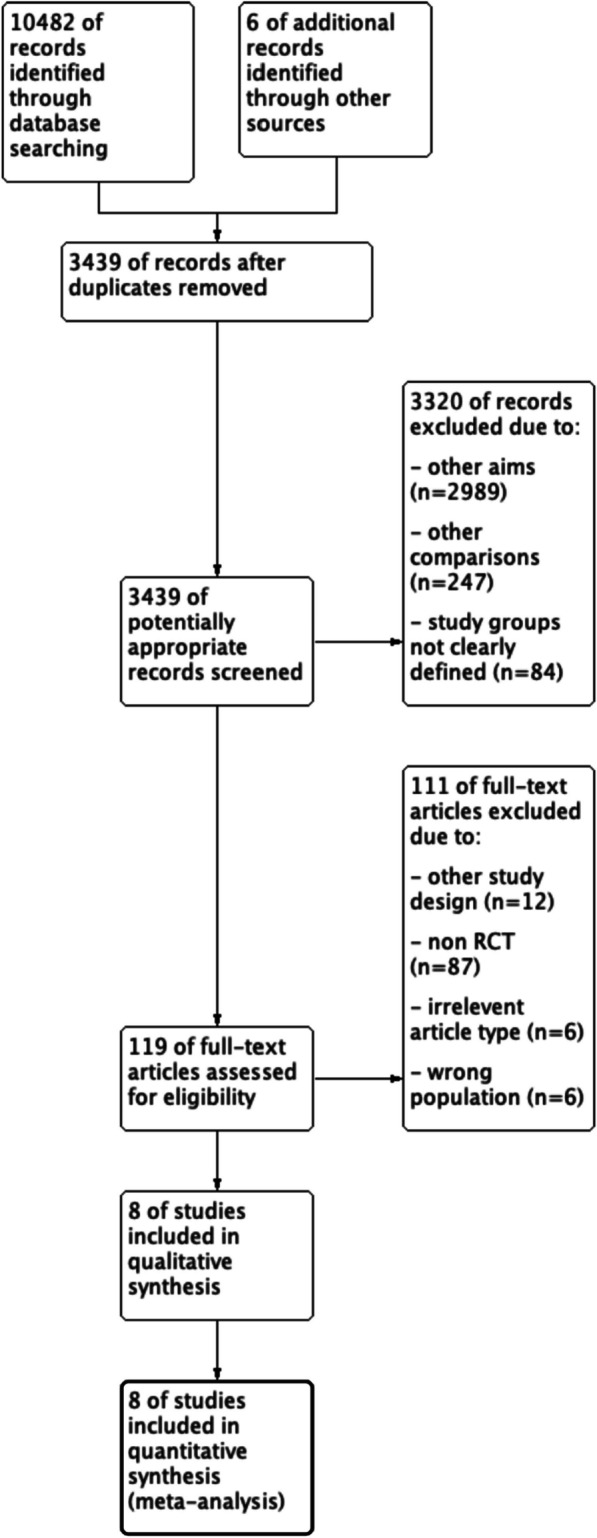
Preferred reporting items for systematic reviews and meta-analysis flow diagram of included randomized control trials in the systematic review and meta-analysis

Figure 1 displays the PRISMA flowchart. Eight RCT fulfilled the inclusion criteria and were included in the meta-analysis (publication dates 1995–2022). In total, 3213 patients were allocated to NOM (*n* = 1615) or OM (*n* = 1598). General characteristics of patients as reported in the studies are shown in Table 1.

**Table 1:** Brief information of the included studies

Study	Study design	Participants	Intervention	Outcomes	Follow-up
Ceresoli [14]	RCT, single center, 45 patients	Participants aged 18–65 years were diagnosed by AIR score and adjunctive abdominal ultrasound in selected participants Participants with intermediate probability of acute appendicitis from AIR score were examined with abdominal ultrasound and were included in the study if ultrasound findings confirmed the clinical suspicion of acute appendicitis Participants with high probability of acute appendicitis from AIR score without signs of perforation and with WCC of less than 15 000/µl and CRP less than 5 mg/l were included for the randomization	Days 1–3: IV ertapenem (1 g, q24h) Days 4–8: PO amoxicillin plus clavulanic acid (1 g TID)"	Primary outcome: resolution of symptoms and inflammatory markers (WCC < 10 000/µl and CRP < 1 mg/l) within 2 weeks after surgery in the surgical group or from the third dose of ertapenem without other treatments in the antibiotic group Secondary outcomes: complications, negative appendicectomy, duration of hospital stay, work absence, long-term negative outcomes within 1 year, including: bowel occlusion/intraperitoneal abscess leading to surgical re-operation, bowel occlusion longer than 48 h, intraperitoneal abscess, incisional hernia or wound dehiscence in the surgical group and recurrence of acute appendicitis in the antibiotic group	
Eriksson [4]	RCT, single center, 40 patients	typical history and clinical signs, positive findings at ultrasound and either increased WCC and CRP values or high CRP or WCC on two occasions within a 4 h interval	Days 1–2: IV cefotaxime (2 g, q12h) plus tinidazole (800 mg, q24h) Days 3–10: PO ofloxacin (200 mg BID) plus tinidazole (500 mg BID)	Pain scores (every 6 h using a visual analogue scale), morphine consumption, WCC and temperature, positive diagnosis at surgery, duration of hospital stay, wound infection and recurrent appendicitis	6, 10, and 30 days AD
Hansson [15]	RCT, multicenter, 369 patients	Participants with positive history, clinical signs, laboratory tests and in some cases, ultrasonography, CT and gynecological examination	Day 1: IV cefotaxime (1 g × 2 doses) plus metronidazole (1.5 g × 1 dose) Days 2–11: PO ciprofloxacin (500 mg BID) plus Metronidazole (400 mg TID)	Treatment efficacy, complications, recurrences and reoperations, duration of antibiotic therapy, abdominal pain after discharge from hospital, duration of hospital stay and sick leave. The total costs for the primary hospital stay were analyzed for each patient	1 month and 1 year AD
Khan [16]	RCT, single center, 130 patients	Participants aged 15–45 years old with positive history, clinical signs, laboratory tests and in some cases, ultrasonography, CT	Days 1–5: ciprofloxacin (250 mg TID) plus metronidazole (500 mg TID), route of administration not specified		
O'Leary [17]	RCT, single center, 186 patients	Participants aged 16 years and older admitted to the emergency department with right iliac fossae pain, raised WCC/CRP, fluent in English (and negative β-HCG in females) were screened for inclusion Participants without exclusion criteria would then proceed to radiological investigation with abdominal ultrasound with/without magnetic resonance imaging performed in those under 45 years; CT in participants above 45 years of age was performed Participants were randomized if acute uncomplicated appendicitis was evidenced from radiological investigation	Intravenous (IV) antibiotic (co-amoxiclav,1.2 g, 3 times daily). IV antibiotics were continued until there was a clinical improvement followed by 5 days of oral co-amoxiclav(625 mg 3 times a day orally for 5 days)	Primary endpoint: success rate of antibiotic treatment at 1-year follow-up for the antibiotic group; successful appendicectomy for the surgical group Secondary endpoints: quality of life, cost and duration of hospital stay	
Salminen [18]	RCT, multicenter, 530 patients	Participants aged 18–60 years admitted to the emergency department with clinical suspicion of acute uncomplicated appendicitis confirmed by CT were considered. Acute appendicitis was considered present when the appendiceal diameter exceeded 6 mm with wall thickening and at least one of the following: abnormal contrast enhancement of the appendiceal wall, inflammatory oedema, or fluid collections around the appendix. Participants with complicated appendicitis, defined as the presence of an appendicolith, perforation, abscess or suspicion of a tumor on the scan, were excluded	Days 1–3: IV ertapenem (1 g/day) Days 4–10: PO levofloxacin (500 mg QD) and metronidazole (500 mg TID)	The primary outcome measure in the antibiotic group was resolution of acute appendicitis, with discharge from hospital without the requirement for surgical intervention and no recurrent appendicitis during the 1-year follow-up Treatment success in the appendicectomy group was defined as the patient successfully undergoing an appendicectomy Secondary outcomes: post-intervention complications, late recurrence of appendicitis (more than 1 year), duration of hospital stay, sick leave taken, pain scores on a visual analogue scale, and the use of analgesics	1 week, 2 months, and 1 year after intervention
Styrud [19]	RCT, multicenter, 252 patients	Men, 18–50 years of age, admitted to six different hospitals between 1996 and 1999. Participants with suspected appendicitis with a CRP concentration above 10 mg/l and with no clinical signs of perforation	Days 1–2: V cefotaxime (2 g, q12h) plus tinidazole (800 mg, q24h) Days 3–12: PO ofloxacin (200 mg BID) plus tinidazole (500 mg BID)	Duration of hospital stay, sick leave, diagnosis at operation, recurrences and complications	1 week, 6 weeks, and 1 year AD
Talan [20]	RCT, single center, 30 patients		Days 1–2: IV ertapenem (1 g/day) Days 3–10: PO cefdinir and metronidazole Dosing is dependent on age Cefdinir: 13 + years, 300 mg BID; 5–12 years, 7 mg/kg BID, max 300 mg Metronidazole: 13 + years, 500 mg tablets TID; 5–12 years, 10 mg/kg TID, max. 500 mg		
The CODA Collaborative [21]	RCT, multicenter, 1552 patients	Consecutive English-speaking or Spanish-speaking participants above 18 years of age were approached by the research coordinator if imaging confirmed they had appendicitis. All participants with evidence of appendicolith from imaging results were included in a prespecified subgroup before randomization. Evidence of perforation from the imaging result was not an exclusion criterion	Day 1: IV metronidazole (+ ceftriaxone or levofloxacin), ertapenem, cefoxitin Days 2–10: PO metronidazole + ciprofloxacin or cefdinir	Primary outcome: 30-day health status, assessed with EQ-5D™ questionnaires Secondary outcomes: appendicectomy in the antibiotics group, patient-reported resolution of symptoms, and National Surgical Quality Improvement Program-defined complications at the time of index treatment or during follow-up, visits to the emergency department or hospital related to appendicitis symptoms, appendiceal neoplasms, treatment-related complications, days of missed work for the participants and their career	
Vons [22]	RCT, multicenter, 239 patients	All adults 18 years and older with suspected acute appendicitis. Eligible participants had CT diagnosis of uncomplicated appendicitis, using defined radiological criteria and were randomized to appendicectomy or antibiotic therapy	IV amoxicillin plus clavulanic acid (3 g/day)	Primary endpoint: occurrence of peritonitis within 30 days of initial treatment, diagnosed either at appendicectomy or postoperatively by CT Secondary endpoints: number of days with a post-intervention visual analogue scale pain score of 4 or higher, duration of hospital stay and absence from work, incidence of complications other than peritonitis within 1 year and recurrence of appendicitis after antibiotic treatment (appendicectomy performed between 30 days and 1 year follow-up, with a confirmed diagnosis of appendicitis)	15, 30, 90, 180, and 360 days AD

### Study characteristics

There was a significant amount of heterogeneity observed among the studies included in the analysis, particularly in terms of the diagnostic criteria used to define uncomplicated appendicitis. Additionally, there was substantial heterogeneity found in the type of antibiotics administered, the duration of administration, and the various outcomes that were evaluated.

### Risk of bias

**Fig. 2:**
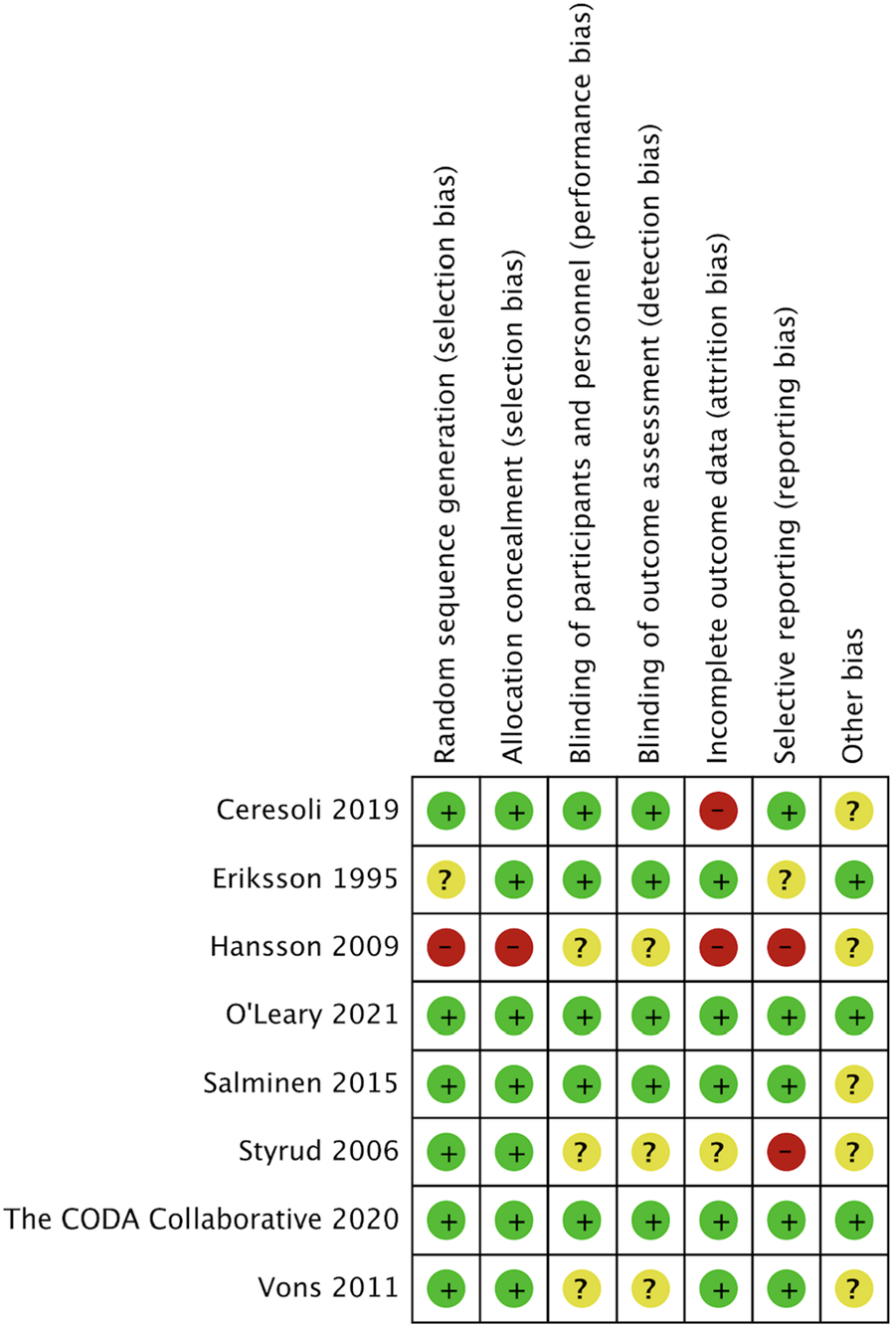
Risk of bias graph of the included studies

Figure 2 shows the RoB (Risk of Bias) analysis, indicating the assessment of bias in the included studies. In terms of study quality assessment, the included RCTs exhibited varying levels of risk across different domains. Out of the 8 RCTs analyzed, 6 studies reported a low risk of selection bias as they adequately described random sequence generation and allocation concealment [14, 17–19, 21, 22]. However, the risk of selection bias remained unclear in two studies, where insufficient information was provided [4, 15].

Concerning attrition bias, two studies were deemed to have a high risk due to inconsistencies in the reported numbers in tables and text [14, 15]. Additionally, two studies were identified as having a high risk of selective reporting due to the lack of predefined endpoints [15, 19].

The meta-analysis portrays a robust picture with most of the included studies exhibiting a low risk of bias across crucial domains. This underscores the reliability of our results, affirming the study's overall credibility.

A graphical representation of the risk of bias assessment is provided in Additional file 1 of the manuscript.

In terms of potential publication bias, no significant indications were observed graphically, as evidenced by the funnel plots. For further details and visual representations, the funnel plots are available as Additional file 1 accompanying this paper.

The risk of language and geographic bias in this study is deemed low, as the nature of the research conducted, and the comprehensive analysis undertaken help mitigate any potential skew toward specific languages or regions.

### Complication-free treatment success (Fig. 3)

**Fig. 3:**
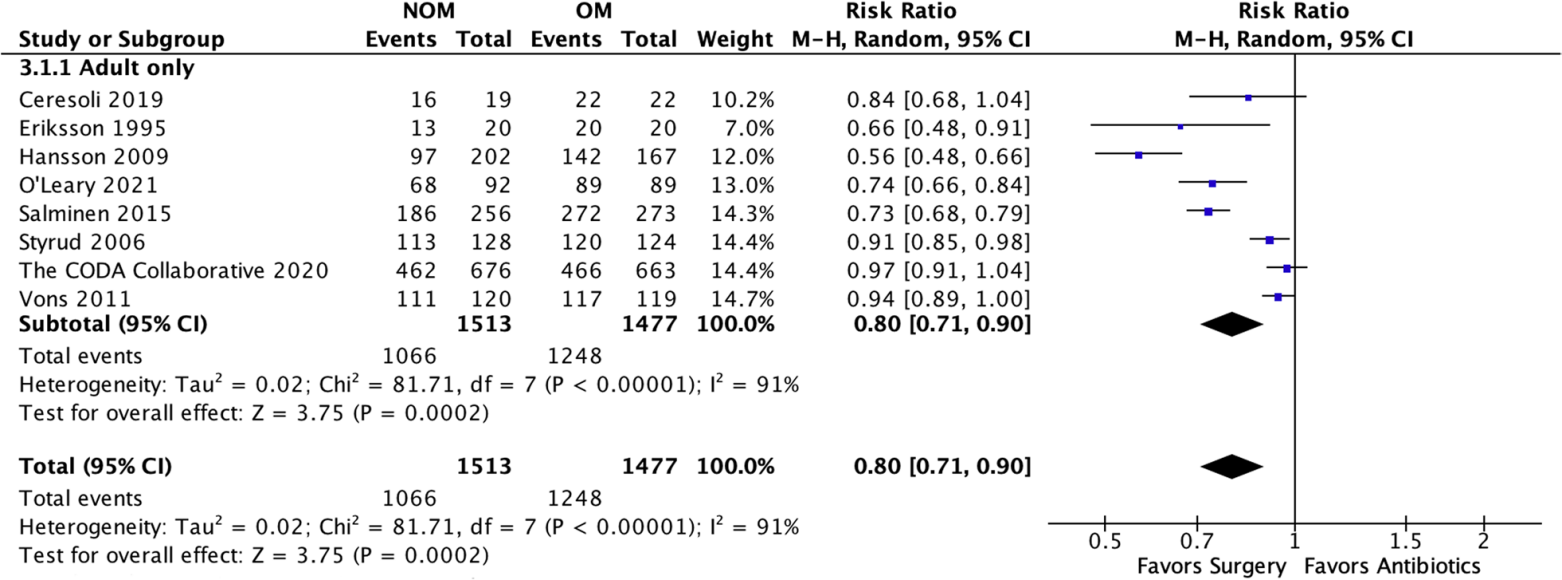
NOM success rate

**Fig. 4:**
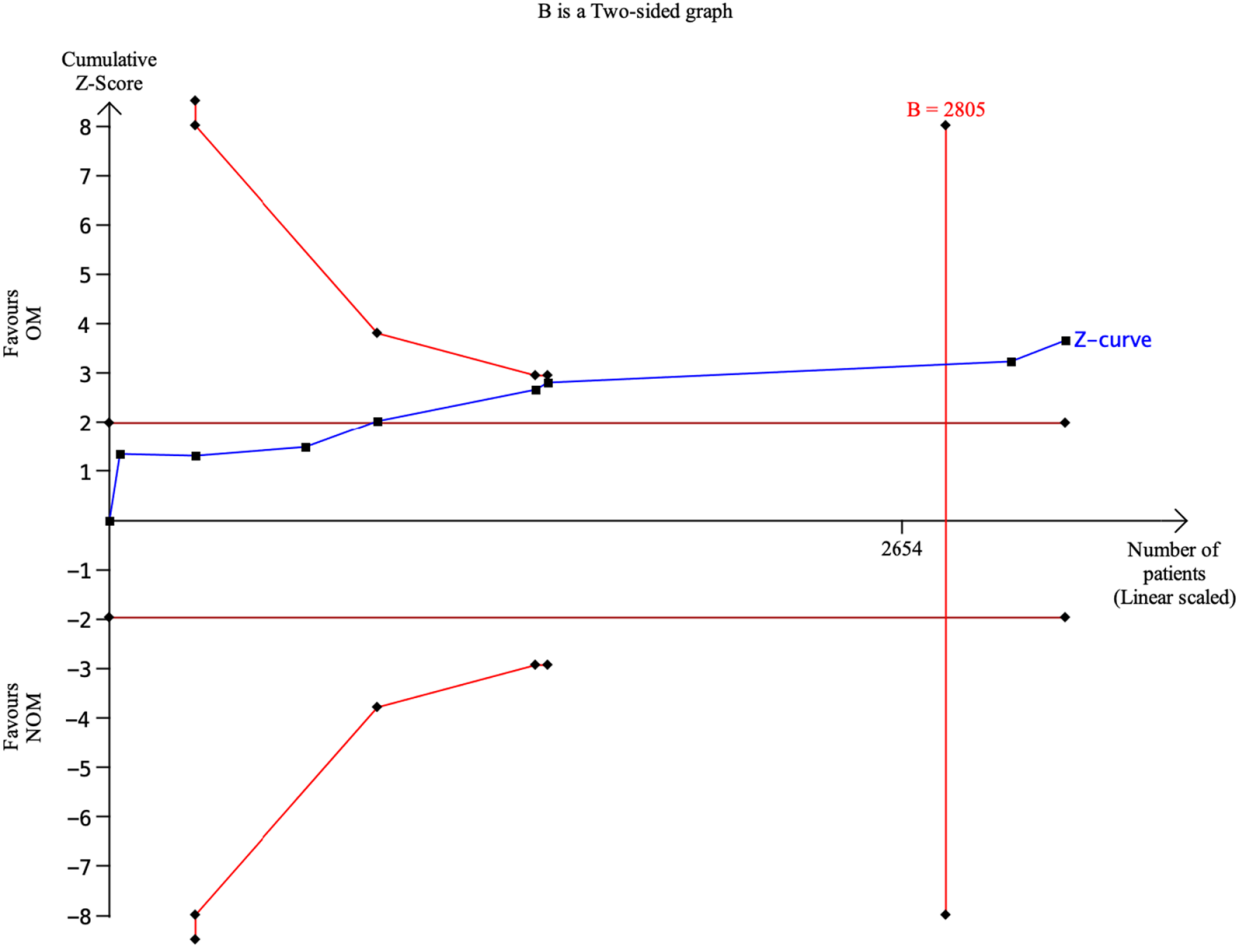
Trial sequential analysis of 8 trials comparing NOM vs. OM for overall treatment efficacy. The cumulative Z-curve crossed the conventional boundary for benefit and required information size but did not cross the trial sequential monitoring boundary for benefit, suggesting that the current evidence is statistically significant but does not support a superiority of OM and further trials will not change this conclusion. A diversity adjusted required information size of 2805 patients was calculated using an alpha = 0.05 (two sided) and a beta = 0.20 (power 80%), and empirical estimation from TSA software

All studies included in the analysis provided data to evaluate the effectiveness of the treatments [4, 14, 15, 17–19, 21, 22]. The results showed that antibiotic treatment had a significantly lower treatment efficacy rate (70.45%, 1066 of 1513) compared to appendectomy (84.49%, 1248 of 1477). The risk ratio (RR) was 0.80 (95% confidence interval 0.71 to 0.90, *p* < 0.00001), indicating a statistically significant difference between the two treatment approaches. Furthermore, a substantial level of heterogeneity was observed in the meta-analysis, with an *I*-squared value of 87%, suggesting significant variation among the included studies. Trial sequential analysis of 8 trials comparing NOM vs. OM for overall treatment efficacy. The cumulative Z-curve crossed the conventional boundary for benefit and required information size but did not cross the trial sequential monitoring boundary for benefit, suggesting that the current evidence is statistically significant but does not support a superiority of OM and further trials will not change this conclusion. A diversity adjusted required information size of 2805 patients was calculated (Fig. 4).

### Treatment efficacy at 1-year follow-up (Fig. 5)

**Fig. 5:**
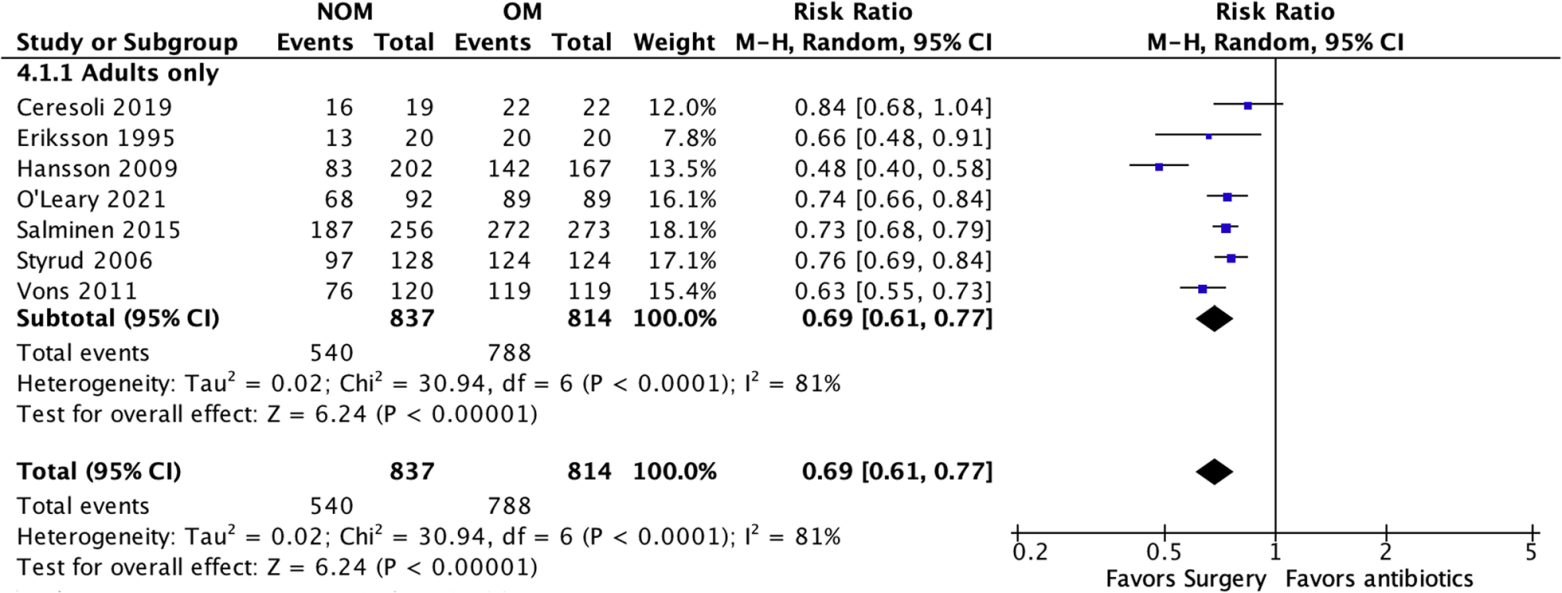
NOM success rate at 1-year follow-up

**Fig. 6:**
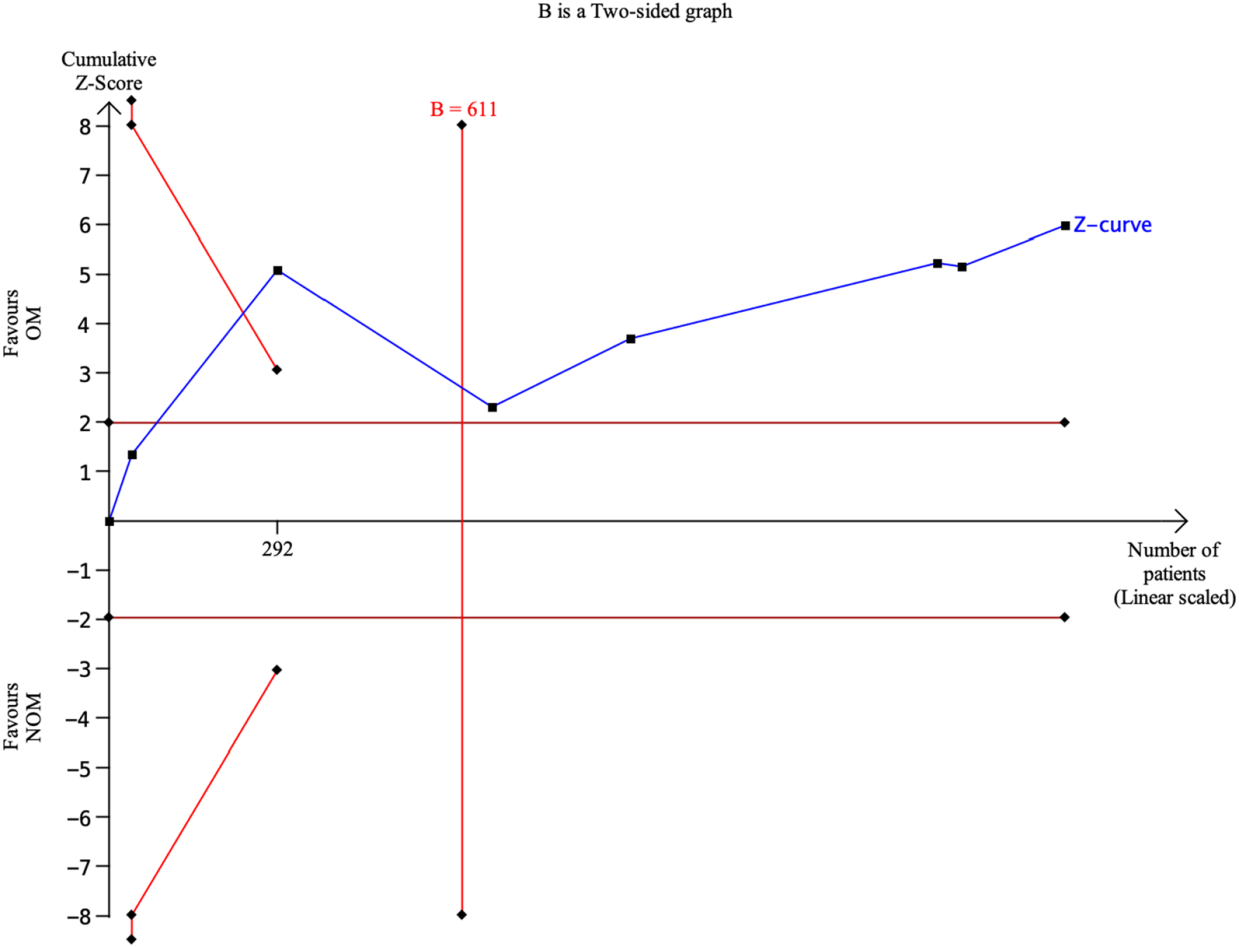
Trial sequential analysis of 7 trials comparing NOM vs. OM for treatment efficacy at 1-year follow-up. The cumulative Z-curve crossed the conventional boundary for benefit, the trial sequential monitoring boundary for benefit and required information size, suggesting that the current evidence is conclusive and further trials will not change this conclusion. A diversity adjusted required information size of 611 patients was calculated using an alpha = 0.05 (two sided) and a beta = 0.20 (power 80%), and empirical estimation from TSA software

Seven studies included in the analysis provided data to evaluate the effectiveness of the treatments at 1-year follow-up [4, 14, 15, 17–19, 22]. The results showed that antibiotic treatment had a significantly lower treatment efficacy rate (64.51%, 540 of 837) compared to appendectomy (96.8%, 788 of 814). The risk ratio (RR) was 0.69 (95% confidence interval 0.61 to 0.77, *p* < 0.00001), indicating a statistically significant difference between the two treatment approaches. Furthermore, a substantial level of heterogeneity was observed in the meta-analysis, with an *I*-squared value of 81%, suggesting significant variation among the included studies. Trial sequential analysis of 7 trials comparing NOM vs. OM for treatment efficacy at 1-year follow-up. The cumulative Z-curve crossed the conventional boundary for benefit, the trial sequential monitoring boundary for benefit and required information size, suggesting that the current evidence is conclusive and further trials will not change this conclusion. A diversity adjusted required information size of 611 patients was calculated (Fig. 6).

### Length of primary hospital stay (Fig. 7)

**Fig. 7:**
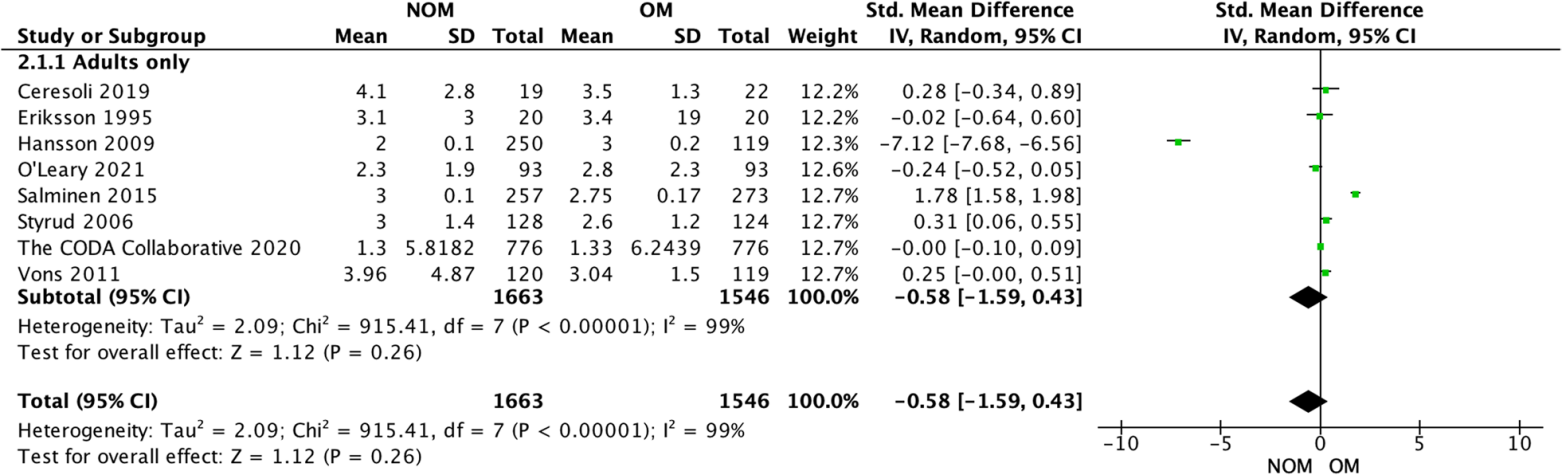
Total length of stay

All studies reported LOS at index hospital admission [4, 14, 15, 17–19, 21, 22]. The analysis showed that there was no statistically significant difference between antibiotic treatment and appendectomy in terms of their effect on the duration of hospital stay. The mean difference was − 0.58 days (95% confidence interval − 1.59 to 0.43, *p* = 0.26), indicating that the difference observed was not statistically significant. However, there was a high level of heterogeneity among the included studies, with an *I*-squared value of 99%, suggesting an important variability in the results across studies.

### Costs (Fig. 8)

**Fig. 8:**
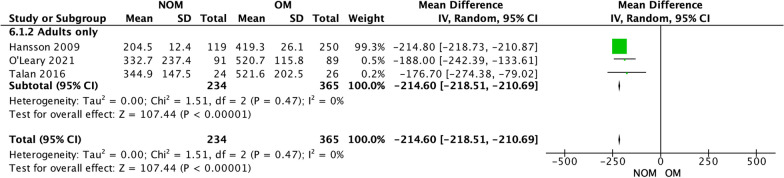
Costs

The pooled analysis of primary costs included 3 studies [15, 17, 20].

Overall, NOM resulted in significantly lower costs when compared to OM (sample size: 599; MD − 214.6; 95% CI − 218.51 − 210.69; *P* < 0.00001, *I*^2^ = 0%). There was a low level of heterogeneity among the included studies, with an *I*-squared value of 0%, suggesting a negligible variability in the results across studies.

### Postoperative complications (Fig. 9)

**Fig. 9:**
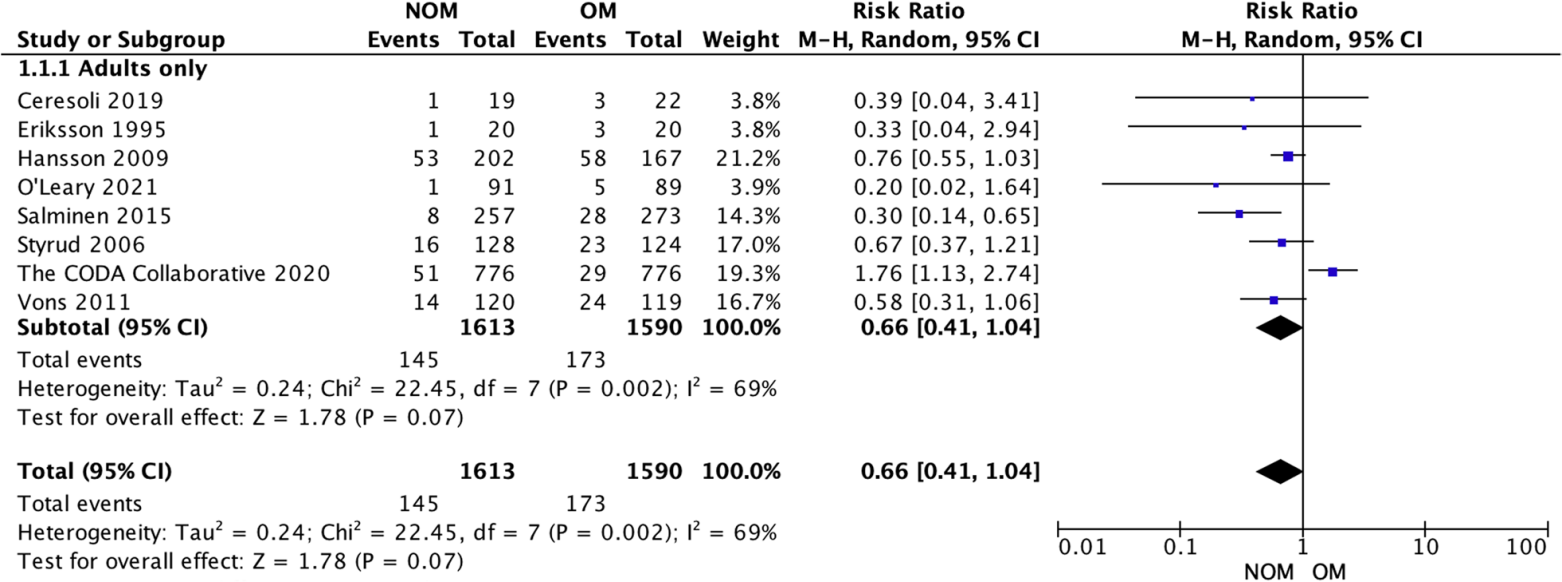
Complications rate

**Fig. 10:**
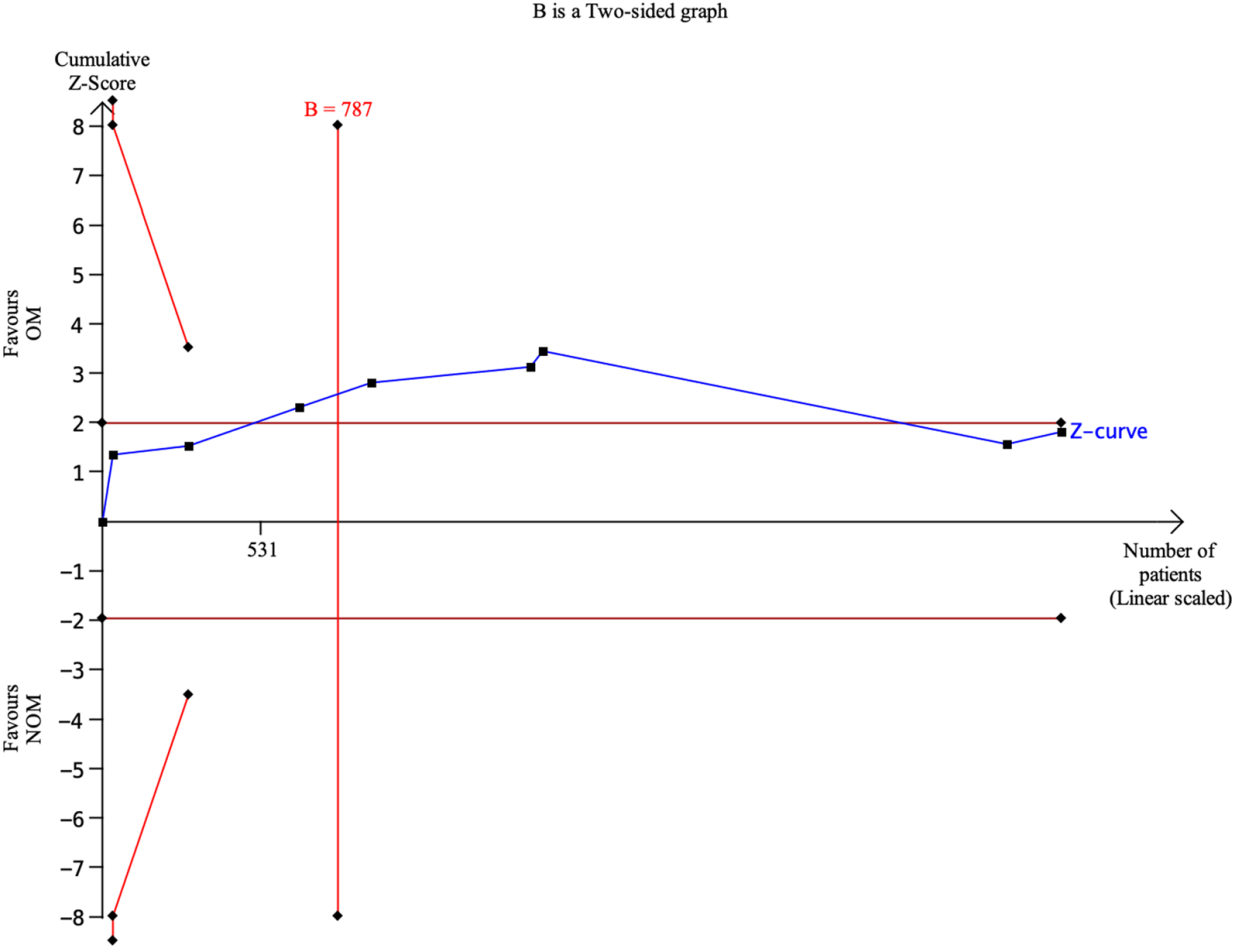
Trial sequential analysis of 8 trials comparing NOM vs. OM for postoperative complications. The cumulative Z-curve did not cross both the conventional boundary and the trial sequential monitoring boundary but crossed the required information size, suggesting that there are no significant differences in terms of complications and further trials difficulty will change this conclusion. A diversity adjusted required information size of 787 patients was calculated using an alpha = 0.05 (two sided) and a beta = 0.20 (power 80%), and empirical estimation from TSA software

Eight studies reported post-treatment complications [4, 14, 15, 17–19, 21, 22]. There was no statistically significant difference in the rate of post-treatment complications between participants treated with antibiotics (8.98%; 145 out of 1613) and those who underwent appendectomy (10.88%; 173 out of 1590). The risk ratio (RR) was 0.66 (95% confidence interval 0.41 to 1.04, *p* = 0.07), indicating that the difference observed was not statistically significant. However, there was a considerable level of heterogeneity among the included studies, with an *I*-squared value of 69%, suggesting some variability in the results across studies. Trial sequential analysis of 8 trials comparing NOM vs. OM for postoperative complications. The cumulative *Z*-curve did not cross both the conventional boundary and the trial sequential monitoring boundary but crossed the required information size, suggesting that there are no significant differences in terms of complications and further trials difficulty will change this conclusion. A diversity adjusted required information size of 787 patients was calculated (Fig. 10).

### Quality of life

Three studies provided data regarding quality of life [17, 20, 21]. However, a pooled analysis could not be done because of numerous scales utilized to evaluate the outcome.

In the Talan et al. trial, NOM patients had higher physical SF-12v2 scores than OM patients at the 2-week and 1-month follow-up intervals (median 54 vs. 44). On the contrary, individuals who had OM both at 2 weeks (median 58 vs 55) and at 1 month follow-up (median 56 vs 55) had higher scores for the mental SF-12v2.

The study “A Randomized Trial Comparing Antibiotics with Appendectomy for Appendicitis” (CODA trial), in a single time point of 30 days following randomization, reported QoL using the EQ-5DTM (EuroQoL Group, Rotterdam, The Netherlands), demonstrating no difference between antibiotic therapy and appendectomy (mean 0.92; SD 0.13 vs. mean 0.91; SD 0.13).

O’Leary et al. assessed quality of life (QoL) using the same scale but at four different points in time (one week, one month, three months, and twelve months after randomization). However, data were reported with participants divided into three groups (appendectomy, antibiotic treatment, and failed antibiotic treatment with subsequent appendectomy). When compared to the group that underwent successful antibiotic therapy, the appendectomy group's mean QoL at 12 months was substantially higher (mean 0.976; CI 0.962 to 0.990 vs. mean 0.888; CI 0.856 to 0.920).

## Discussion

This study, including 3213 patients and 8 RCTs [2, 4, 14, 15, 17–24], is, to our knowledge, the largest meta-analysis of randomized controlled trials conducted thus far encompassing an adult population.

The results demonstrate that antibiotic therapy as a first-line treatment has a failure rate of 29.5% during the initial hospitalization, 35.4% at 1-year follow-up, a non-statistically significant difference in terms of length of stay (LOS), a comparable rate of complications and significantly lower costs compared to surgical treatment.

Several meta-analyses over the previous years have highlighted that surgical treatment is associated with an increased rate of complications, such as the study by Podda et al. [25], published in 2019. On the contrary, two recent studies [6, 26] did not observe a lower rate of complications in the conservatively treated group. Our study aligns with these latter findings. This is likely attributed to the higher number of laparoscopic appendectomies performed more recently. As compared to open technique, laparoscopic appendectomy has been shown to significantly reduce wound infection rates [27]. In our analysis the rate of laparoscopic appendectomies performed was 68.44%, as reported by 6 RCTs. Furthermore, recent trials included in our study predominantly analyzed laparoscopic appendectomies, with a percentage of 100% for a trial [14], 96% [21], and 90% [17], respectively. In contrast, previous studies, particularly the Antibiotic Therapy vs Appendectomy for Treatment of Uncomplicated Acute Appendicitis (APPAC) trial and the study conducted by Styrud et al., primarily consisted of open procedures.

Another important factor that could influence these results is the presence of appendicoliths. In the CODA trial, participants who were randomized to antibiotic medication and had an appendicolith experienced problems with a rate of 14% compared to 2% in those who did not [21]. This latter trial and the study by Vons et al. [22] included patients with appendicoliths diagnosed by CT scan. The other trials had a heterogeneous diagnostic protocol, so several patients with appendicoliths may have remained unrecognized.

In conclusion, we can affirm that NOM is safe, as it has a comparable rate of complications to laparoscopic appendectomy. However, there was heterogeneity in diagnostic assessment, antibiotic regimens and treatment duration among the various studies, which could impact the results.

The higher number of laparoscopic appendectomies may have also influenced the outcome regarding LOS. It is well-established in the literature that LOS is shorter when the procedure is performed laparoscopically, leading to an equivalence in LOS with conservative treatment [27]. It was not possible to perform a subgroup analysis due to lack of the necessary data. However, it would be important, in the future, to have RCTs that perform totally laparoscopic appendectomies, as Ceresoli et al. did, or that perform a subgroup analysis to explore the differences between laparoscopic and laparotomy appendectomies for this outcome.

However, this result is certainly influenced by significant heterogeneity in the implementation and management of conservative therapy. Indeed, there is inconsistency among the studies regarding the type of antibiotic used, the duration of intravenous administration, and subsequent oral administration. For example, in the CODA trial [21], an initial bolus, administered on the first day, was followed by oral therapy from the second to the tenth day. On the other hand, O’Leary et al. [17] continued that the antibiotic therapy until a clear clinical improvement of the patient was achieved.

Regarding the results concerning the complication-free treatment success during the initial hospitalization, they significantly favor surgical intervention. The conservative treatment has an efficacy rate of 71.84% in the index hospitalization.

Undoubtedly, a clear advantage of appendectomy is the ability to remove the pathogenic cause with a negligible risk of stump appendicitis [28]. Conversely, this is not possible with conservative treatment, which carries a significant risk of lifetime recurrence, estimated between 6.7% and 8.6% [29].

The treatment effectiveness assessed at one-year follow-up demonstrates a greater effectiveness of surgery compared to conservative treatment; this latter has an efficacy of 67.3% at one year compared to 97.4% for appendectomy.

It is important, therefore, to determine whether a conservative treatment with a lower efficacy measured at one-year follow-up and with comparable rates of complications, can be considered acceptable and feasible as a first-line treatment. It is true that approximately one-third of patients experience a recurrence within the first year. However, according to the 5-year follow-up results of the APPAC trial, patients can be successfully treated again with antibiotic therapy, and if surgery is required, it does not appear to be associated with increased complications or technical difficulty.

In fact, when Salminen et al. [30] published the 5-year follow-up findings of the APPAC randomized clinical trial in 2018, they addressed the issue of the paucity of research on the long-term clinical efficacy of antibiotics, which had previously been seen as one of the most significant barriers to the widespread adoption of NOM for uncomplicated appendicitis. Only 2.3% of patients undergoing surgery for recurrent appendicitis were found to have complicated forms of the disease and the overall complication rate was significantly lower in the antibiotic group than in the appendectomy group (6.5% vs. 24.4%, *P* = 0.001) among patients who were initially treated with antibiotics for uncomplicated appendicitis.

Recently, Pàtkovà et al. have published a cohort study regarding the long-term outcomes of NOM [31]. This study drew patients from two RCTs included in this meta-analysis: Eriksson et al.'s study [4] published in 1995 and Styrud et al.'s study [19] published in 2006. The article concludes that over the course of two decades, more than half of the patients treated through NOM did not experience recurrences, and there is no evidence of long-term risks associated with NOM, except for the recurrence itself. The long-term follow-up confirmed the feasibility of NOM as a surgical alternative. It would be very important to have new RCTs that analyze the results of the comparison between NOM and OM in the long term, in order to draw more robust conclusions on the topic.

Therefore, given these circumstances, an informed patient choice is crucial, in our opinion. In a study published by Hanson et al. [32] in 2018, 9.4% of the surveyed population responded that they would opt for nonoperative management (NOM) in the case of appendicitis. This number increased to 14.5% when asked about choosing for their children. The study focused on discussing the failure rates of NOM, and indeed, the authors themselves speculate that different numbers would have been obtained if the success rates were presented to patients. A more recent study, published in 2021 by Bom et al., presents very different results. Approximately half of the participants in the average population sample expressed a preference for antibiotics as a treatment for uncomplicated appendicitis, even if it entailed a higher risk of recurrence, in order to avoid surgery initially. Additional rigorous qualitative research will be necessary to investigate the factors behind the strikingly different outcomes observed in these two studies and to gain a deeper understanding of patient preferences in various situations.

We are faced with two therapies that are equivalent in terms of safety, with one being less expensive, less effective, and non-invasive, while the other is more expensive, more effective, and invasive. Beyond the decision of which therapy should be considered first-line, the outcome that could matter the most is the patient's quality of life.

Regarding this latter outcome, the diversity of presented results highlights the need for more literature. To establish more reliable analyses, it is crucial to use homogeneous scales across various trials. It is interesting to notice, despite the limitations outlined above, that in the three studies examined in one case, there is no difference in QoL between NOM and OM, and in the remaining two, the surgery appears to be associated with higher QoL.

Interpreting these results for clinical application requires consideration of several limitations. The significant heterogeneity limits confidence, variations in intervention expertise and the broad timespan of included RCTs may introduce confounding factors. Our study encompassed RCTs spanning a significant time frame from 1995 to 2022. Over this period, there were significant advancements in surgical techniques, diagnostic imaging, and antibiotic selection, resulting in noticeable variations in treatment protocols across the included studies. These variations were evident in the use of different antibiotics and the progression of surgical techniques from predominantly open appendectomies to primarily laparoscopic procedures throughout the chronology of the included RCTs. These variations have resulted in a high degree of heterogeneity among the studies, which constitutes a significant limitation, potentially biasing the analysis.

## Conclusions

This meta-analysis and trial sequential analysis provide evidence that NOM with antibiotics is safe and, in the majority of cases, successful. NOM is equivalent to surgery in terms of complications and LOS while also incurring lower costs. While NOM's efficacy is lower than surgery, it does not seem to increase long-term complications. In relation to the three primary outcomes examined in our study, the evidence gleaned from current literature can be regarded as conclusive. It is highly unlikely that new RCTs focusing on these outcomes will substantially alter the existing body of evidence available to date. Thus, offering NOM and discussing its risks and benefits with the patient is reasonable based on this data.

Further scientific efforts should be directed toward the attempt to provide surgeons with tools that allow the early identification of those patients who might respond adequately to NOM.

### Supplementary Information

**Additional file 1.** Supplementary Figures 1–6.

## Data Availability

Data-sharing requests will be considered by the management group upon written request to the corresponding author. If agreed, deidentified participant data will be available, subject to a data-sharing agreement.
