# Supplementary material for: A meta-analysis and trial sequential analysis comparing nonoperative versus operative management for uncomplicated appendicitis: a focus on randomized controlled trials
Source: World J Emerg Surg. 2024 Jan 13;19:2. doi: 10.1186/s13017-023-00531-6 (PMC10787963; doi:10.1186/s13017-023-00531-6)
Supplement: Supplementary file 1 — Additional file 1. Supplementary Figures 1–6. [file 13017_2023_531_MOESM1_ESM.docx]

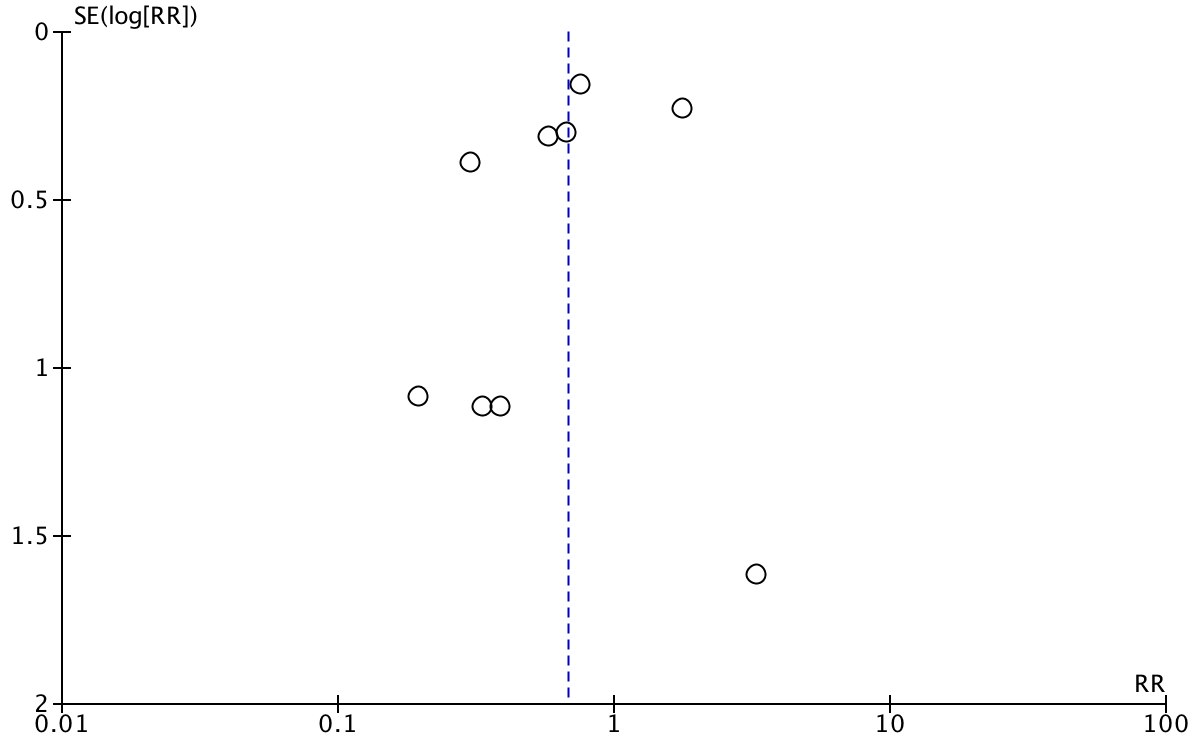

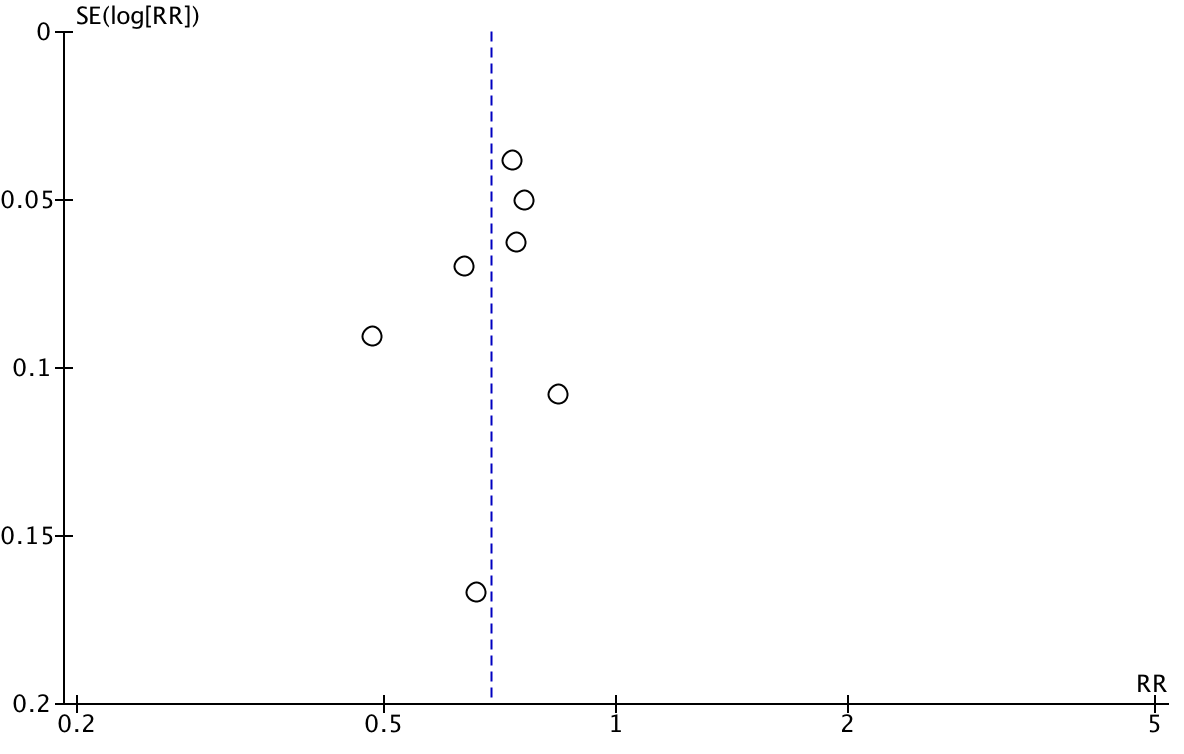


Fig. 2e: Funnel plot, complications NOM vs. surgery

Fig. 1e: Funnel plot success rate at 1 year NOM vs. surgery


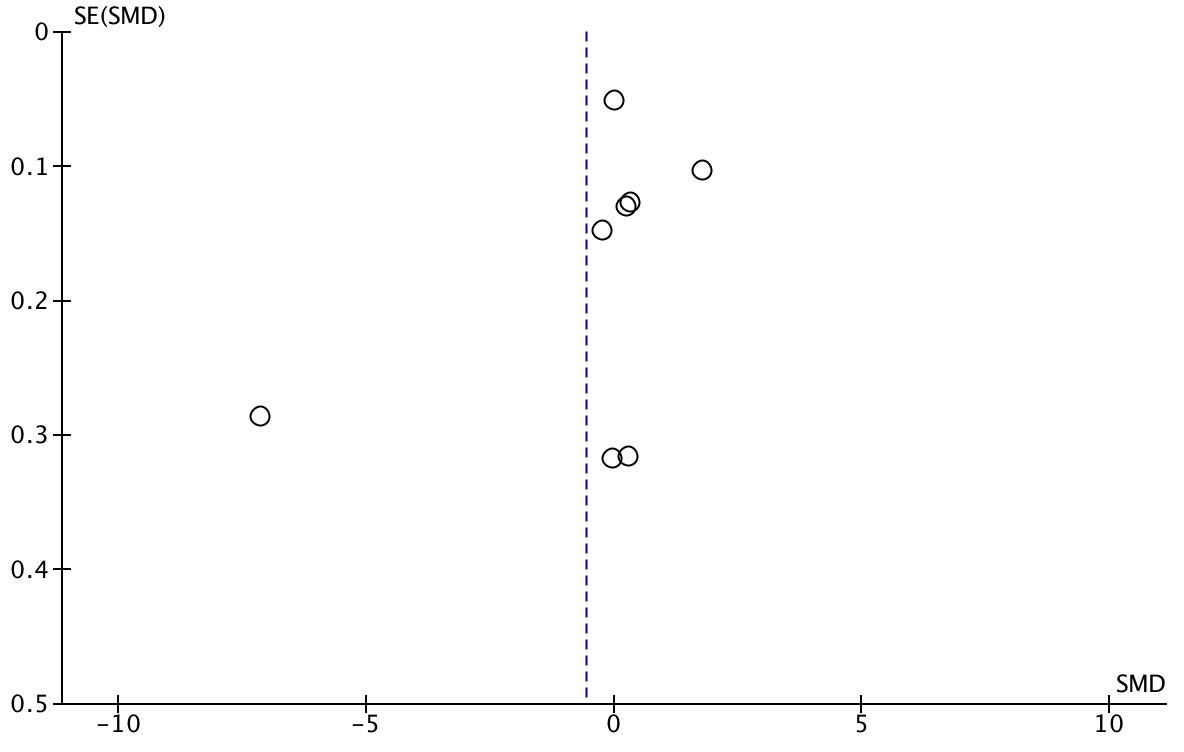

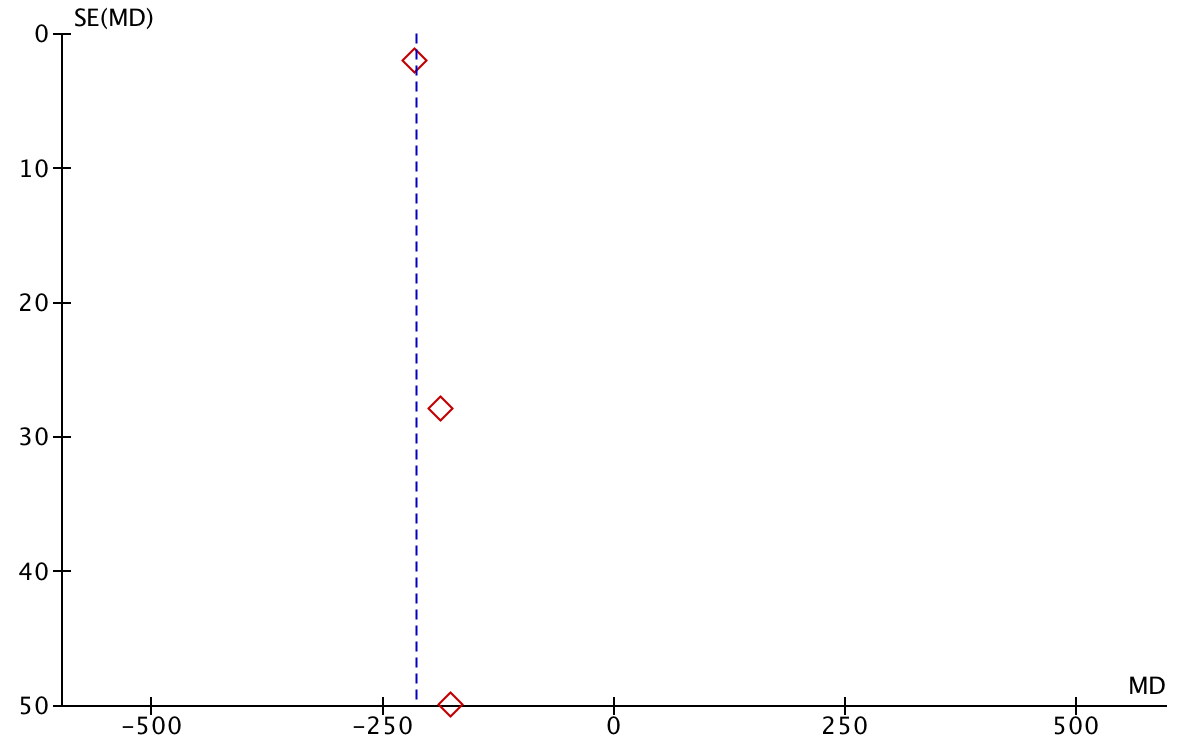


Fig. 4e: Funnel plot, LOS NOM vs. surgery

Fig. 3e: Funnel plot, costs NOM vs. surgery


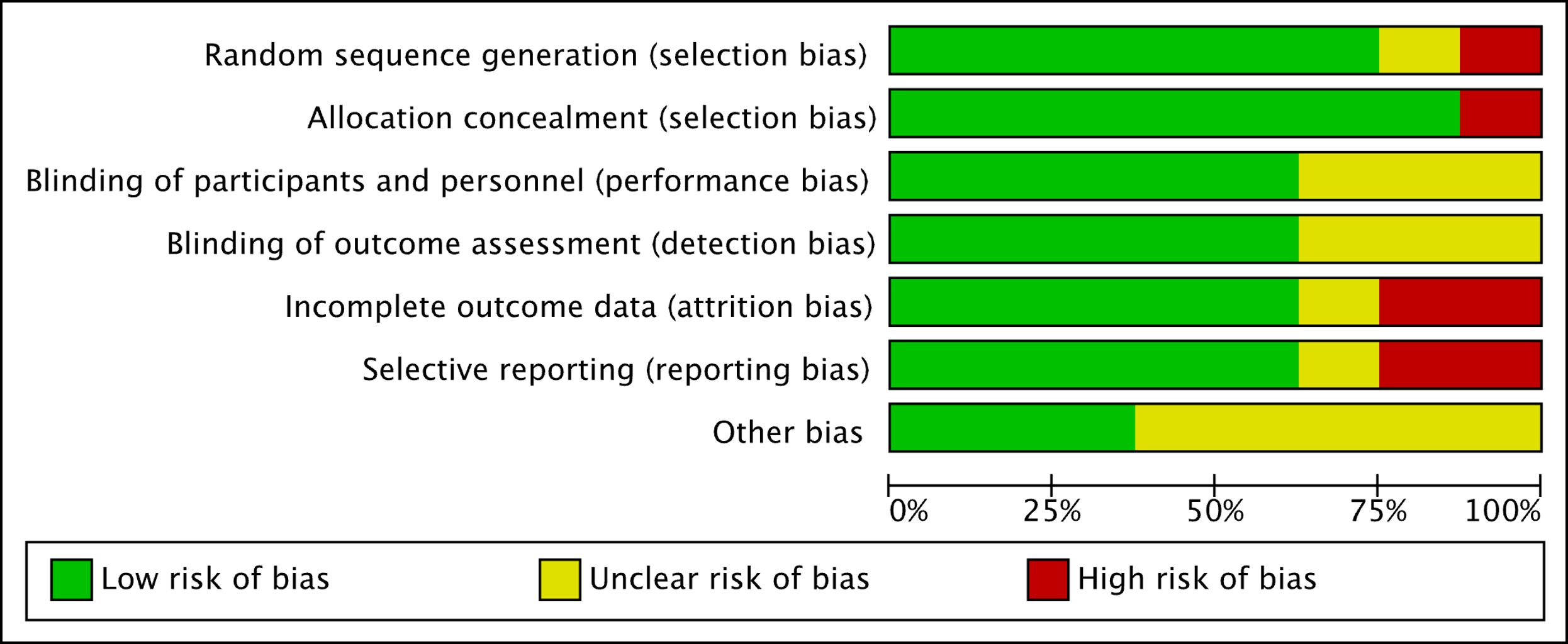

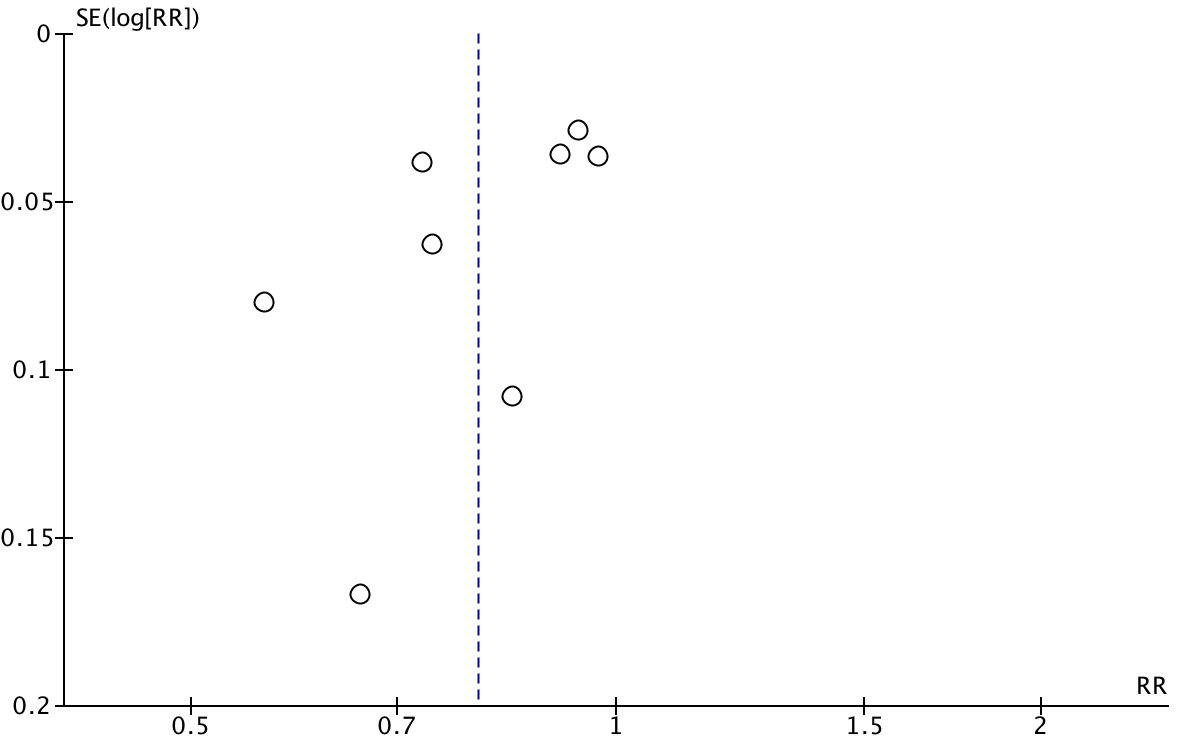


Fig. 6e: Risk-of-bias summary: review authors’ judgments about each risk-of-bias item for included studies.

Fig. 5e: Funnel plot overall success rate NOM compared to surgery
